# Analysis of Gene Coexpression by B-Spline Based CoD Estimation

**DOI:** 10.1155/2007/49478

**Published:** 2007-03-13

**Authors:** Huai Li, Yu Sun, Ming Zhan

**Affiliations:** 1 Bioinformatics Unit, Branch of Research Resources, National Institute on Aging, National Institutes of Health, Baltimore, MD 21224, USA

## Abstract

The gene coexpression study has emerged as a novel holistic approach for microarray data analysis. Different indices have been used in exploring coexpression relationship, but each is associated with certain pitfalls. The Pearson's correlation coefficient, for example, is not capable of uncovering nonlinear pattern and directionality of coexpression. Mutual information can detect nonlinearity but fails to show directionality. The coefficient of determination (CoD) is unique in exploring different patterns of gene coexpression, but so far only applied to discrete data and the conversion of continuous microarray data to the discrete format could lead to information loss. Here, we proposed an effective algorithm, CoexPro, for gene coexpression analysis. The new algorithm is based on B-spline approximation of coexpression between a pair of genes, followed by CoD estimation. The algorithm was justified by simulation studies and by functional semantic similarity analysis. The proposed algorithm is capable of uncovering both linear and a specific class of nonlinear relationships from continuous microarray data. It can also provide suggestions for possible directionality of coexpression to the researchers. The new algorithm presents a novel model for gene coexpression and will be a valuable tool for a variety of gene expression and network studies. The application of the algorithm was demonstrated by an analysis on ligand-receptor coexpression in cancerous and noncancerous cells. The software implementing the algorithm is available upon request to the authors.

## 

[1,2,3,4,5,6,7,8,9,10,11,12,13,14,15,16,17,18,19,20,21,22,23,24,25,26,27,28,29]
